# Management of Adverse Reactions to Loncastuximab in Patients With Relapsed or Refractory Diffuse Large B‐Cell Lymphoma

**DOI:** 10.1002/hon.70128

**Published:** 2025-08-20

**Authors:** Narendranath Epperla, Adam J. Olszewski, Emily C. Ayers, Sairah Ahmed

**Affiliations:** ^1^ Division of Hematology and Hematologic Malignancies Huntsman Cancer Institute University of Utah Salt Lake City Utah USA; ^2^ Division of Hematology and Oncology Brown University Health Providence Rhode Island USA; ^3^ Division of Hematology and Oncology University of Virginia Health System Charlottesville Virginia USA; ^4^ Department of Lymphoma/Myeloma and Stem Cell Transplantation & Cellular Therapy UT MD Anderson Cancer Center Houston Texas USA

**Keywords:** adverse events, cutaneous reactions, diffuse large B‐cell lymphoma, effusion, loncastuximab tesirine

## Abstract

Diffuse large B‐cell lymphoma (DLBCL) is the most common B‐cell non‐Hodgkin lymphoma in the world. Treatment options for relapsed DLBCL in the third line and beyond include chimeric antigen receptor T‐cell therapy, T‐cell–engaging bispecific antibodies, and loncastuximab tesirine (loncastuximab tesirine‐lpyl [Lonca]), each with unique toxicity profiles. There is still an unmet need for guidance on managing Lonca‐associated adverse events (AEs), particularly for oncologists who have limited experience with this antibody–drug conjugate. Here, an online survey among lymphoma specialists in the US between June and August 2024 assessed practice patterns and experiences, including Lonca treatment patterns, AE management, patient concerns, and physician perceptions. Based on these responses, an algorithm was developed to help manage Lonca‐associated AEs. The most commonly reported AEs were edema and rash/photosensitivity, typically occurring within the first 4 doses, whereas fatigue was the most common patient concern. Lonca‐associated AEs were managed by delaying or discontinuing Lonca or by prescribing diuretics, steroids, or antihistamines, depending on the AE observed. The survey results align with findings from prior clinical trials and support the manageability of Lonca‐associated AEs in a wide variety of settings. The included algorithm provides guidance for managing AEs, such as edema, myelosuppression, and cutaneous reactions.

## 1 Introduction

Diffuse large B‐cell lymphoma (DLBCL) is the most common aggressive type of non‐Hodgkin lymphoma, accounting for approximately 31% of adult cases [1, 2]. The standard first‐line treatments for DLBCL include regimens such as rituximab, cyclophosphamide, doxorubicin, vincristine, and prednisone (R‐CHOP) or pola‐R‐CHP (wherein vincristine is replaced by polatuzumab vedotin), which have cure rates of approximately 53% to more than 70% [3, 4, 5, 6, 7]. However, approximately 40% of patients with DLBCL have disease that eventually relapses following first‐line treatment [8].

For patients with relapsed or refractory (R/R) DLBCL, second‐line treatment options include chimeric antigen receptor T‐cell therapy (CAR‐T) (i.e., axicabtagene ciloleucel and lisocabtagene maraleucel) for patients with primary refractory disease or DLBCL that relapses within 1 year of first‐line therapy [3, 9, 10]. For subsequent lines of treatment, options include cellular therapies, such as CAR‐T; T‐cell–engaging bispecific antibodies; antibody–drug conjugates (ADCs), such as loncastuximab tesirine (loncastuximab tesirine‐lpyl [Lonca]) and polatuzumab‐vedotin; monoclonal antibody combinations (i.e., tafasitamab plus lenalidomide); small‐molecule inhibitors, such as selinexor; and combination therapies, including lenalidomide/rituximab/brentuximab vedotin [3, 11, 12, 13, 14, 15, 16, 17, 18]. In patients with late relapses (> 1 year following the completion of first‐line chemoimmunotherapy), autologous hematopoietic cell transplantation could be considered should they achieve chemosensitivity [19, 20]. Despite all the available treatments, challenges remain with existing regimens, including high relapse rates and an unmet need for new therapeutic options. There is a need for targeted therapies with manageable adverse event (AE) profiles that can be used in the community setting and for older and/or less fit patients.

Lonca is an ADC comprised of a humanized anti‐CD19 antibody conjugated via a linker region to a pyrrolobenzodiazepine (PBD) dimer cytotoxin [21, 22]. Upon binding to CD19, Lonca is internalized, the linker is cleaved, and the PBD dimers are released [22, 23]. The PBD dimers bind to DNA and form cytotoxic DNA crosslinks, which lead to blocked cell division and subsequent cancer cell apoptosis [22, 23]. Lonca received accelerated approval from the United States (US) Food and Drug Administration in April 2021 as a single agent for the treatment of adult patients with R/R DLBCL after two or more lines of systemic therapy, including for DLBCL not otherwise specified, DLBCL arising from low‐grade lymphoma, and high‐grade B‐cell lymphoma [24].

Lonca's pivotal, open‐label, single‐arm, Phase 2 LOTIS‐2 trial (NCT03589469) included 145 participants with R/R DLBCL, had a median follow‐up of 7.8 months, an overall response rate (ORR) of 48.3% (95% CI: 39.9–56.7), and a complete response rate of 24.8% [12, 25]. In the LOTIS‐2 trial, treatment‐emergent adverse events (TEAEs) considered likely to be related to the PBD payload included edema or effusion (31% of patients), skin or nail‐related AEs (43%), and liver enzyme abnormalities (51%), with PBD‐related AEs generally being grade 1 or 2 in severity [12]. Most patients were able to continue treatment, with some requiring dose delays. Patients received oral dexamethasone premedication to prevent PBD‐related toxicities (i.e., edema and rash) unless contraindicated and were advised to avoid prolonged sun exposure and to report potential skin rashes. To manage AEs, treatment delays of up to 5 weeks and dose reductions were allowed, and patients with weight gain over 1 kg from day 1 of cycle 1 or with edema/pleural effusions were treated with spironolactone [12].

Updated safety analyses demonstrated that an all‐grade TEAE occurred in 98.6% of patients, and TEAEs occurring in ≥ 30% of the all‐treated population were increased gamma‐glutamyl transferase (GGT; 42%), neutropenia (40%), and thrombocytopenia (33%) [25]. Grade ≥ 3 TEAEs were reported in 73.8% of patients, and those reported in ≥ 10% of the all‐treated population were neutropenia (26%), thrombocytopenia (18%), increased GGT (17%) and anemia (10%). TEAEs leading to treatment discontinuation occurred in 24.8% of patients in the all‐treated population, with the most common being GGT (12.4%), peripheral edema (2.8%), localized edema (2.1%), and pleural effusion (2.1%). No increase in toxicity was observed in older patients compared with younger patients [25].

Real‐world studies have been conducted to investigate Lonca utilization and treatment settings [26, 27]. A study of 118 patients with R/R DLBCL who received third‐line Lonca following second‐line CAR‐T (*n* = 95) or fourth‐line Lonca following third‐line CAR‐T (*n* = 23) demonstrated an ORR of 73% and 78%, respectively [26]. A retrospective chart review presented data from 187 patients with R/R DLBCL from 21 US centers who had received Lonca as a second‐ or third‐line (19%) or fourth‐line and beyond (81%) therapy [27]. Most of the patients had received prior CAR‐T (60%), but only 16% had received prior autologous stem cell therapy. AEs were documented in 35% of patients with AEs of interest, including cytopenias (17%), peripheral edema (11%), rash (10%), pleural effusion (3%), and pericardial effusion (0.5%). Treatments reported after Lonca therapy included immunochemotherapy (31%), tafasitamab‐lenalidomide (17%), and CAR‐T (10%) [27].

Despite increasing data availability, there remains an unmet need for comprehensive guidance on Lonca's current usage patterns, clinical management, and Lonca‐associated AEs, particularly for oncologists practicing in the community setting who may have limited clinical experience with Lonca. This article provides clinical practice recommendations for managing AEs commonly associated with Lonca treatment in patients with R/R DLBCL based on the real‐world experience of clinicians who have adopted Lonca into their practice and gained expertise in practical management of its AEs.

## 2 Materials and Methods

### 2.1 Survey Development

An online questionnaire was developed to assess the practice patterns and clinical experience of hematologists using Lonca in the treatment of R/R DLBCL. The survey was distributed via email to lymphoma specialists at academic institutions located across the US. The survey contained 51 questions, 44 of which were quantitative, offering single or multiple‐choice responses, while 7 were qualitative, allowing for open‐ended feedback. The questionnaire and its administration plan were designed and reviewed by the authors.

The survey covered several key domains, beginning with respondent characteristics, which included age, gender, race/ethnicity, geographic location, and practice details to assess the representativeness of the respondents. Lonca treatment patterns were explored, including its sequencing with other therapies, such as CAR‐T, autologous/allogeneic transplantation, and bispecific antibodies. Factors influencing treatment initiation, duration, and discontinuation were also investigated. The AE patterns and patient counseling domain focused on identifying the most common and severe AEs associated with Lonca, the number of doses before AE onset, and which AEs led to dose reductions, delays, or discontinuations. Additionally, the questionnaire explored how physicians counseled patients regarding potential AEs. Specific questions addressed the management of Lonca‐associated AEs, including prophylactic dexamethasone use for edema/effusion and the occurrence of peripheral edema, pleural or pericardial effusions, myelosuppression, and cutaneous reactions, such as rashes and photosensitivity. The final domain examined common patient concerns and physician perceptions of Lonca's safety profile.

### 2.2 Study Sample

The study targeted oncologists specializing in the treatment of DLBCL, who are currently in clinical practice. To be included in the full analysis, respondents were required to practice oncology within the US and have prior experience prescribing Lonca.

### 2.3 Recruitment of Respondents

The online survey was administered via the survey website SurveyMonkey (San Mateo, CA) between June 24, 2024, and August 16, 2024. No financial incentives were provided, and pilot testing estimated the survey completion time to be approximately 30 min.

### 2.4 Ethics Approval and Consent to Participate

The study was conducted in compliance with the Declaration of Helsinki. Given the nature of the study (survey), this study was exempt from Institutional Review Board requirements, and informed consent was waived.

### 2.5 Data Analysis

Summary descriptive analyses, including mean and median values with ranges, were calculated for survey responses. No statistical significance tests were conducted. Respondents not currently treating patients with Lonca were included in the respondent characteristics analysis but excluded from Lonca use–related analyses.

For Lonca sequencing, responses of “never” were categorized as no, while other responses (i.e., “rarely,” “sometimes,” “often,” and “most of the time”) were categorized as yes. Free‐response qualitative answers were grouped when applicable. For questions on AEs leading to Lonca dosage adjustments, bone marrow suppression‐related responses (i.e., myelosuppression, cytopenia, and neutropenia) were grouped, as were those related to fluid retention (i.e., edema and third spacing).

## 3 Results

### 3.1 Respondent Characteristics

Sixteen physicians responded to the survey. The demographic and practice details of respondents are listed in Table 1. A substantial proportion of respondents identified as female (31%) and non‐White (31%). Most of the respondents had been in practice for 6 or more years post‐fellowship (69%). The respondents were from practices across various regions of the US, including the Northeast (i.e., Connecticut, Rhode Island, New York, and Pennsylvania), South (i.e., Virginia, North Carolina, Florida, and Texas), Midwest (i.e., Ohio and Missouri), and West (i.e., Colorado, Utah, and California). Most respondents practiced at universities or academic hospitals (94%) with the ability to administer CAR‐T and/or stem cell transplants.

**TABLE 1:** Respondent characteristics.

Characteristic	Survey respondents (*N* = 16)
Sex, *n* (%)	
Male	10 (62.5)
Female	5 (31.3)
Did not to answer	1 (6.3)
Race/ethnicity, *n* (%)	
White	9 (56.3)
Asian	4 (25.0)
Hispanic	1 (6.3)
Did not answer	2 (12.5)
Years in practice post‐fellowship, *n* (%)	
≤ 5 years	5 (31.3)
6–10 years	7 (43.8)
> 10 years	4 (25.0)
Practice setting, *n* (%)	
University/academic hospital	15 (93.8)
Community practice associated with an academic hospital	1 (6.3)
Practice type, *n* (%)	
Nontransplant, lymphoma‐focused practice	6 (37.5)
Combined lymphoma and transplant/cell therapy practice	10 (62.5)
Cellular therapy capability at practice, *n* (%)	
CAR‐T therapy and transplant	15 (93.8)
Transplant only	1 (6.3)
Number of patients with DLBCL seen in each line of therapy in the last year, *mean (range)*	
First line	25 (8–60)
Second line	20 (1–55)
Third line or later	18 (0–50)
Number of patients with DLBCL treated with Lonca, *n* (%)	
1–5	7 (43.8)
6–10	5 (31.3)
11–20	2 (12.5)
21–30	1 (6.3)
> 30	0
Unsure	0
Have never used Lonca	1 (6.3)

Abbreviations: CAR‐T, chimeric antigen receptor T‐cell therapy; DLBCL, diffuse large B‐cell lymphoma; Lonca, loncastuximab tesirine.

In total, 44% of respondents reported treating between 1 and 5 patients with DLBCL with Lonca (Table 1). Respondents reported seeing an average of 25 DLBCL patients in the first‐line setting (range, 8–60 patients), 20 patients in second‐line setting (range, 1–55 patients), and 18 patients in third‐line setting or later (range, 0–50 patients) in the last year. Of the 16 respondents, 15 (94%) had experience with Lonca and were included in the full‐response analysis.

### 3.2 Lonca Treatment Patterns

**FIGURE 1:**
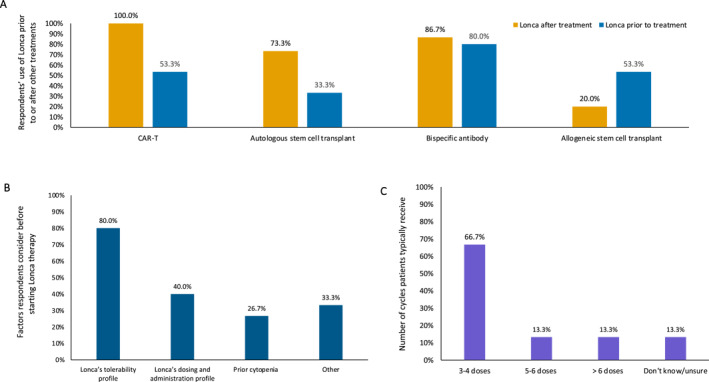
Lonca treatment patterns, including (A) the use of Lonca to treat relapsed/refractory DLBCL before or after other treatments, (B) factors respondents considered before starting Lonca therapy, and (C) the number of Lonca cycles the typical patient received. For panel A, responses of “rarely,” “sometimes,” “often,” and “most of the time” were combined into yes. CAR‐T, chimeric antigen receptor T‐cell; DLBCL, diffuse large B‐cell lymphoma; Lonca, loncastuximab tesirine.

Lonca was used by the respondents in a variety of settings, both before and after other CD19‐directed therapies, and both on‐label and off‐label (e.g., as a second‐line bridging therapy), highlighting the versatility of the use of the agent. More respondents indicated having used Lonca after versus before CAR‐T (100% vs. 53%, respectively), autologous stem cell transplants (73% after vs. 33% before), and bispecific antibodies (87% after vs. 80% before) (Figure 1A). In contrast, more respondents indicated using Lonca before versus after allogeneic stem cell transplants (53% vs. 20%).

Respondents indicated that the most common considerations before choosing Lonca treatment were Lonca's tolerability profile (80%), Lonca's dosing and administration protocol (40%), and the patients' prior occurrences of cytopenia (27%). Other considerations (33%) included the patients' fitness and CD19 status, the efficacy of Lonca, the potential for using Lonca in patients with existing effusions, and the option for a clinical trial or other treatment (Figure 1B). Most respondents (67%) stated their patients typically received 3 to 4 Lonca doses (Figure 1C). Respondents indicated that unplanned discontinuations of Lonca occurred more commonly due to disease progression (80%) than because of AEs (20%).

### 3.3 Adverse Event Patterns and Patient Counseling

**FIGURE 2:**
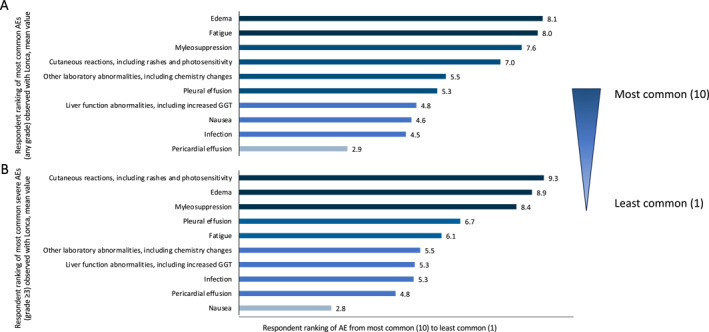
Respondent rating of (A) the most common adverse events of any grade or (B) the most common severe adverse events of any grade observed with using Lonca. Respondents ranked the adverse events from the most common (value of 10) to the least common (value 1) and were instructed to select “N/A” if they had not observed the adverse event with Lonca in their practice. AE, adverse event; GGT, gamma‐glutamyl transferase; Lonca, loncastuximab tesirine; N/A, not applicable.

Respondents indicated edema was the most common AE (any grade) and that cutaneous reactions (including rashes and photosensitivity) were the most common severe AEs (grade ≥ 3) observed with Lonca in their clinical experience (Figure 2). Other commonly reported AEs included fatigue, myelosuppression, pleural effusion, and other laboratory abnormalities, including chemistry changes. For patients who experienced a Lonca‐associated AE, respondents indicated that over a third experienced the AE after 1 to 2 doses (45% of patients) or 3 to 4 doses (45%).

Respondents indicated that when Lonca doses were reduced or held, myelosuppression/neutropenia was the most common reason for reducing (33%) or holding (33%) the Lonca dosage (Table 2). Respondents routinely provided counseling to patients receiving Lonca regarding sunlight exposure/protective measures (93%), shortness of breath (93%), fever or bruising/bleeding (80%), and periodic monitoring of blood counts (60%).

**TABLE 2:** Most common adverse events associated with Lonca dosage being reduced, held, or discontinued.^a^

	Survey respondents (*N* = 15)
Most common reasons for Lonca dosage to be reduced, *n* (%)	10 (66.7)
Myelosuppression/neutropenia	5 (33.3)
Edema/third spacing^b^	3 (20.0)
Cutaneous reaction/rash	2 (13.3)
Most common reasons for Lonca dosage to be held, *n* (%)	14 (93.3)
Myelosuppression/neutropenia	5 (33.3%)
Edema/third spacing^b^	4 (26.7)
Cutaneous reaction/rash	4 (26.7)
Effusion	1 (6.7)
Most common reasons for Lonca dosage to be discontinued, *n* (%)	11 (73.3)
Pleural/pericardial effusion	3 (20.0)
Progressive disease	3 (20.0)
Cutaneous reaction/rash	2 (13.3)
Edema/third spacing^b^	2 (13.3)
Myelosuppression/neutropenia	1 (6.7)

Abbreviations: AE, adverse event; Lonca, loncastuximab tesirine.

^a^ Respondents were asked to indicate the primary reason for reducing, holding, or discontinuing Lonca dosage; as the question was a free‐response question, if 2 adverse events were provided, only the first was counted.

^b^ Category excludes pleural and pericardial effusions, which were collected separately.

### 3.4 Lonca‐Associated Adverse Events

**FIGURE 3:**
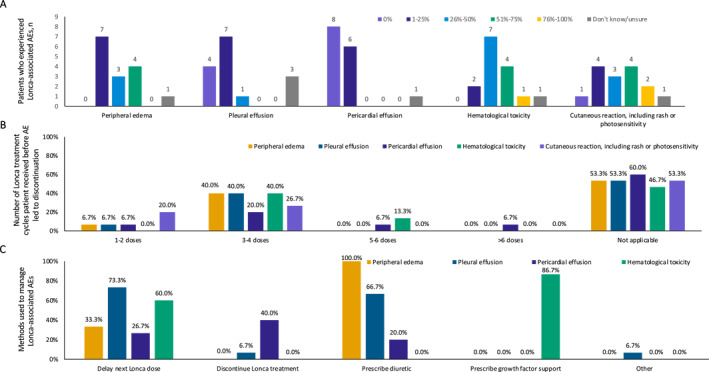
Respondent ratings of Lonca‐associated AEs, including (A) the percentage of patients who experienced Lonca‐associated AEs, (B) the number of Lonca treatment cycles before an AE led to discontinuation, and (C) the treatments used to manage AEs. For panel C, diuretics were not listed as a treatment option for Lonca‐associated hematological toxicity, but growth factor support was. Growth factor support was the only treatment option provided for hematological toxicity. Cutaneous reactions, such as rash or photosensitivity, were not plotted because their management options (i.e., antihistamines, topical steroids, systemic steroids) differed from those of other conditions. AE, adverse event; Lonca, loncastuximab tesirine.

Most respondents (93%) indicated that the majority (i.e., 76%–100%) of their patients were treated with prophylactic dexamethasone to manage edema/effusion. In response to being asked to estimate how often they encountered typical Lonca‐associated AEs, respondents indicated that peripheral edema was common, with most indicating it was observed in ≤ 50% of their patients and over a quarter indicating it was observed in > 50% of their patients (Figure 3A). In contrast, pleural and pericardial effusion were relatively rare, with approximately 73% and 93% of respondents, respectively, reporting ≤ 25% incidence of those AEs. When discontinuation of Lonca was required, it typically occurred after 3 to 4 doses of Lonca and was in response to peripheral edema, pleural effusion, hematological toxicity, cutaneous reactions, or pericardial effusion (Figure 3B).

When delaying a Lonca dose was necessary, respondents indicated that common reasons included management of pleural effusion, hematological toxicity, peripheral edema, or pericardial effusion (Figure 3C). All respondents prescribed diuretics to manage peripheral edema; over half selected either spironolactone (60%) or loop diuretics (60%). When managing Lonca‐associated cutaneous reactions, most respondents used topical steroids (87%), followed by H2 blocking antihistamines (53%), H1 blocking antihistamines (47%), or systemic steroids (13%).

### 3.5 Patient Concerns and Physician Perceptions

The most common patient concerns reported by physicians were fatigue (47%) and photosensitivity (27%). Most respondents indicated that they thought Lonca has a comparable safety profile with other treatments for R/R DLBCL (60%), with just over a quarter indicating they thought Lonca has a more tolerable safety profile (27%), and one respondent indicating it was less tolerable (13%).

## 4 Discussion

The aim of this study was to offer clinical recommendations for managing AEs in patients receiving Lonca for R/R DLBCL based on input from US oncologists specializing in lymphoma. The survey results on Lonca safety and AE management were consistent with prior LOTIS trials, and respondents provided views that were generally aligned with the LOTIS trials, but with additional insights based on experiences outside of the LOTIS clinical trials in the era of widespread availability of CAR‐T and bispecific antibody therapies.

Physicians reported selecting Lonca both before and after bispecific antibodies. It should be noted, however, that the approval of bispecific antibodies in the US for DLBCL occurred only toward the middle of 2023 [16, 17]. The optimal sequencing of bispecific antibodies in relation to other treatments, including CAR‐T, is still being studied. Some oncologists may prefer to use bispecific antibodies as a bridging therapy prior to CAR‐T, in combination with other therapies, as the next line of treatment after CAR‐T, or as an alternative for patients who progress after, are ineligible for, or lack access to CAR‐T [28, 29].

**FIGURE 4:**
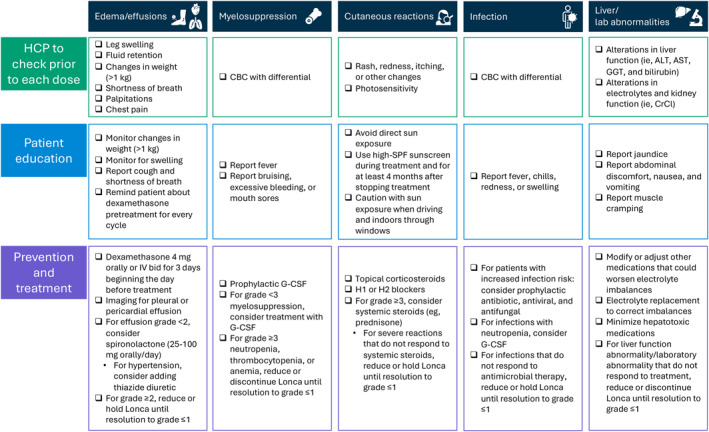
Lonca treatment algorithm, including recommendations for patient monitoring and treatment strategies for oncologists. ALT, alanine aminotransferase; AST, aspartate aminotransferase; bid, twice daily; CBC, complete blood count; CrCl, creatinine clearance; G‐CSF, granulocyte colony–stimulating factor; GGT, gamma‐glutamyl transferase; HCP, healthcare practitioner; IV, intravenous; Lonca, loncastuximab tesirine; SPF, sun protection factor.

To assist oncologists and other healthcare practitioners (HCPs) with the identification and management of Lonca‐associated AEs, we have developed an algorithm for managing the most common Lonca‐associated AEs, including edema, effusions, myelosuppression, cutaneous reactions (including rashes and photosensitivity), infections, along with liver function and laboratory abnormalities (Figure 4). Most Lonca‐associated AEs appear relatively early during treatment (i.e., within 4 doses) with higher Lonca dosages and may abate once the Lonca dosage is reduced for subsequent cycles. In addition, as Lonca‐associated AEs are mostly predictable and easily identifiable, oncologists and advanced practice providers (i.e., registered nurse practitioners and physician assistants) can administer a focused, symptom‐based assessment of common symptoms, such as weight gain, swelling, fatigue, shortness of breath, and rash. As edema, effusions, myelosuppression, and rash are the primary reasons for Lonca dose delays or discontinuations, early intervention by HCPs using standardized protocols is recommended.

Specifically for edema and effusions, we recommend the consideration of spironolactone with an initial dose (i.e., 25–100 mg/day orally) based on edema severity and titrated as clinically indicated, with a potential addition of other agents (i.e., thiazide or loop diuretics) for more severe edema, hypertension, or in patients at risk for hyperkalemia. For cutaneous reactions, we recommend monitoring for common reactions (i.e., rash, redness, and itching) and patient education on common concerns (i.e., itching, photosensitivity, and sun exposure). For sun exposure, we recommend counseling patients to avoid direct sun exposure and use high sun‐protection factor sunscreen for at least 4 months after discontinuing Lonca to allow for drug clearance. Case studies have demonstrated that when managing cutaneous reactions, including photosensitivity rash, antihistamines (H1 and H2 blockers) and topical corticosteroids (i.e., clobetasol) can be used to diminish associated immune responses [30]. For more severe cutaneous reactions that do not respond to topical steroids and/or that have spread to other areas of the body, we recommend considering systemic steroids (i.e., prednisone taper starting at 0.5 mg/kg daily) and reducing or holding Lonca until symptom resolution [31]. Also, we recommend alerting patients about the use of dexamethasone before treatment to prevent PBD‐related toxicities (i.e., edema and rash). For infection management, we propose the use of a growth factor (i.e., granulocyte colony–stimulating factor) for patients with infection secondary to neutropenia. Across all these AEs, patient education is important, so patients can recognize and report relevant symptoms to allow for early treatment intervention.

This study has several limitations, including responses predominantly from academic centers, which may affect the generalizability of the findings as the usage pattern of Lonca and clinical management may vary between the academic and community settings. Additionally, responses were somewhat qualitative in nature, and the ranges of patient experience were broad (e.g., 44% of respondents reporting treating 1 to 5 DLBCL patients with DLBCL using Lonca), so the percentages may not accurately reflect the actual clinical prevalence of Lonca‐associated AEs. However, the study has notable strengths, including the collection of responses across a diverse selection of respondents located in the US and input from actively practicing oncologists specializing in DLBCL, ensuring that the feedback comes from experts in lymphoma management.

## 5 Conclusion

Based on the results of a survey of US oncologists specializing in lymphoma, including DLBCL, Lonca‐associated AEs are manageable in a variety of clinical settings. This aligns with prior real‐world data which have also demonstrated that Lonca can be administered effectively in community practices [26]. The survey findings were consistent with prior LOTIS clinical trials, indicating the tolerability of Lonca in real‐world practice. Respondents provided largely similar opinions on the management of key AEs, such as edema, effusions, myelosuppression, and cutaneous reactions, indicating a similar approach across different settings. Overall, we found that Lonca‐associated AEs can be effectively managed by oncologists practicing in the community setting, supporting its broader use in clinical practice. This article provides a comprehensive overview of Lonca use and AE management and serves as a quick reference for oncologists planning to treat patients diagnosed with R/R DLBCL with Lonca.

## Author Contributions

N.E., A.J.O., S.A., and E.C.A. contributed to the development and distribution of the survey. All authors were involved in the design and formulation of the survey questions and ensured its appropriate dissemination to the target audience. N.E. led the coordination of survey distribution across various centers, while A.J.O., S.A., and E.C.A. provided critical input in refining the survey content. The authors did not receive compensation for their contributions to the survey. All authors participated in the interpretation of the survey results, as well as the revision of the manuscript. Finally, all authors reviewed and gave final approval of the submitted manuscript.

## Ethics Statement

The study was conducted in compliance with the Declaration of Helsinki. Given the nature of the study (survey), this study was IRB exempt.

## Consent

Informed consent was waived.

## Conflicts of Interest

N.E.: Research support to the institution for clinical trials from BeiGene, Incyte, Lilly, Ipsen, ADC Therapeutics; Ad boards for Ipsen, Genentech, and CRISPR Therapeutics. A.J.O.: Clinical research scholar of the Leukemia and Lymphoma Society; reports consultancy role with Genmab, Schrodinger, Blue Cross and Blue Shield of Rhode Island, Bristol‐Myers Squibb, and ADC Therapeutics; and receives research funding from Adaptive Biotechnologies. E.C.A.: Research funding from Abbvie, ADC Therapeutics, Regeneron, and Lilly. Consultancy for ADC Therapeutics and Bristol‐Myers Squibb. S.A.: Research support to institution for clinical trials from Nektar, Merck, Xencor, Chimagen and Genmab; has membership on Chimagen scientific advisory committee; serves on data safety monitoring board for Myeloid Therapeutics; and is a consultant for ADC Therapeutics and KITE/Gilead.

## Peer Review

The peer review history for this article is available at https://www.webofscience.com/api/gateway/wos/peer-review/10.1002/hon.70128.

## Data Availability

Data is available upon request to the corresponding author within the limits of the participant confidentiality.
